# Emerging source of infection – *Mycobacterium tuberculosis* in rescue dogs: a case report

**DOI:** 10.1099/acmi.0.000168

**Published:** 2020-09-07

**Authors:** Silja Mentula, Veera Karkamo, Teresa Skrzypczak, Jaana Seppänen, Hanne-Leena Hyyryläinen, Marjo Haanperä, Hanna Soini

**Affiliations:** ^1^​ Department of Health Security, Finnish National Institute for Health and Welfare, Helsinki, Finland; ^2^​ Veterinary Bacteriology and Pathology Unit, Finnish Food Authority, Helsinki, Finland

**Keywords:** dogs, imported infections, *Mycobacterium tuberculosis*, pathologists, public health, veterinarians

## Abstract

Rescue dog activity is a heavily increasing form of dog charity. Imported homeless dogs represent a reservoir of zoonotic diseases putting owners, veterinarians and pathologists repeatedly at risk. The clinical signs of tuberculosis in a dog are non-specific and diagnosis is often delayed or dismissed. We present a case of 9 months of possible exposure at home and definite exposure at laparotomy and autopsy to intestinal tuberculosis in a family dog imported from Romania to Finland. Persistent gastrointestinal symptoms started 2 years after the import. Abdominal pain, diarrhoea and vomiting proceeded and led to spontaneous death. *Mycobacterium tuberculosis* was identified in the liver, lymph nodes and intestine at autopsy. Exposed persons were notified and follow-up was provided, and no further infections were identified within 12 months of follow-up. The heavily increasing import of companion animals presents unexpected public health risks, such as prolonged exposure to tuberculosis, of which the general public is not aware. The dramatic consequences and high costs of tuberculosis could be reduced through accessible information of the risks of imported animals to both the general public and veterinarians, in addition to availability of rapid diagnostics and proper personal protection.

## Introduction

Rescue dog activity is a form of emerging dog charity, where neglected or abandoned dogs are placed in a new home, often in another country. Its popularity is increasing and hundreds of thousands of dogs are relocated within Europe yearly, of which several tens of thousands come to Finland [1]. In addition to charity and animal rights aspects, rescue dogs are available at a reasonable price, compared to dogs from other sources. In Finland, most rescue dogs originate from Spain, Greece, Russia and Romania.

The risk that these dogs represent a reservoir of zoonotic diseases is not fully recognized. At risk are primarily the owners and attending veterinarians and pathologists. Additionally, in the case of *Mycobacterium bovis *, the spread of bovine tuberculosis (TB) to countries previously free of the disease could cause significant economic losses in cattle and dairy farming. Risks may be unexpected, especially when the incidences of different zoonotic diseases or antibiotic resistance rates differ significantly between the original and the receiving country. Therefore, veterinarians may not be familiar with symptoms of a locally rare disease and diagnostics may not be widely available. As import is also a form of business, the operations may have unethical features; for example, the vaccination certificates may not be valid, i.e. no detectable serological sign of vaccination [2] or discrepancies in locations between documents, such as local vaccine availability [3]. In addition is the problem of smuggling. Together with the original country of the animal, the circumstances and conditions in which the dog has lived directly affect the risks for different diseases.

In spite of the unquestionable progress in the fight against human and bovine TB, human TB is still very prevalent in certain areas, including Middle Africa and Southeast Asia, with dense populations and poor social conditions. In Europe, the incidence of TB is generally low (mean 25.9, range 4.5–72.0, cases per 100 000 in EU/EEA, 2017), but the burden of multidrug-resistant TB is the highest in the world (mean 18 %, range 0–22 %, of new cases) (www.euro.who.int). East European countries, such as Bulgaria, Latvia, Lithuania and Romania, are among the World Health Organization-defined high-TB-priority countries with incidences of 25, 32, 50 and 72 per 100 000, respectively. In Finland the incidence is 4.9 per 100 000. TB in dogs is uncommon, and dogs are not considered a relevant source of transmission or the true risk is unknown [4]. However, *Mycobacterium tuberculosis* and *M. bovis* can infect dogs with similar signs and lesions as in other hosts. Dogs are usually infected by eating contaminated sputum, milk or tissue, or by inhaling contaminated aerosols [5]. Canine infections caused by *M. bovis* are probably related to infected cattle or unpasteurized milk or their by-products, while *M. tuberculosis* infections are mostly respiratory infections of human origin [4]. When infected via ingestion, the main infection focus is the intestines and abdominal cavity. As humans are the only reservoir host for *M. tuberculosis *, and dogs have prolonged close contacts with humans, aerosol exposure from an infected owner is the most likely transmission route for *M. tuberculosis* in a family dog. Therefore, *M. tuberculosis* infection in dogs may be termed an ‘anthropozoonosis’ [5]. There are a limited number of reports of sporadic *M. tuberculosis* cases in dogs, and in all of them the most likely source of infection was a human companion [6–11]. In these cases the infected organs varied between lungs, lymph nodes, intestine, liver, kidneys, spleen and heart, and the disease was most often disseminated.

In dogs, the clinical signs of TB are non-specific, such as weakness, weight loss, fever and coughing, depending on the organs affected and the severity of the infection. Lesions are polymorphic. TB infection may attack any organs and mimic other disorders, so clinical signs alone are not diagnostic. Radiographs and a thorough epidemiological history are useful. TB is confirmed by microscopy and culture or nucleic acid amplification, most often after autopsy. A tuberculin test or serology is not recommended due to possibly unreliable results [5]. Defining the extent and progression of the disease may be difficult. Diagnosis is often delayed and treatment of the contagious dog takes several months, and therefore treatment is generally not recommended. Moreover, the human TB treatment regimen is not directly adaptable to dogs. Isoniazid causes neurological side effects, rifampicin is hepatotoxic, streptomycin is reserved for human use only, and *M. bovis *, which is the most common cause of TB in dogs, is resistant to pyrazinamide [12, 13]. Animals may also have to be euthanized despite treatment [3], suboptimal therapy may select drug resistance, and the risk for recurrence and re-infection remain, if exposure is repeated [14]. These cases therefore pose serious public health safety concerns, and the general resolution is that affected family dogs should be euthanized.

This report describes a case of intestinal *M. tuberculosis* infection diagnosed after autopsy in an imported rescue dog with symptoms starting 2 years after import.

## Case report

The present case was a 5-year-old crossbred spayed female dog weighing 14 kg, born in 2013 and imported from Romania to Finland at the beginning of 2015. The first owner in Finland had several other dogs and cats. The owner changed after 6 months. The second owners had no other pets, but were frequently visited by their four children and several grandchildren. The dog was seemingly healthy until January 2018, when it first showed persistent gastrointestinal symptoms, including abdominal pain and diarrhoea, followed later also by vomiting. No respiratory symptoms were recorded. Dietary changes, and tylosin and metrodinazole treatments were tried.

Hypoalbuminaemia and hyperglobulinaemia were diagnosed and cortisone was prescribed in April. Later an abdominal ultrasound scan was performed, and masses were seen in the liver and the intestines. They both were biopsied on laparascopy in June. On histological examination, acid-fast bacilli (Ziehl–Neelsen stain) and opportunistic yeasts morphologically consistent with *Histoplasma capsulatum* (GSM stain) were detected, and serology for histoplasmosis was positive. Anti-yeast drug therapy was prescribed. Although the condition of the dog fluctuated over the following months, the gastrointestinal symptoms persisted and worsened, and the dog died spontaneously in October 2018, without any respiratory symptoms, and was sent to autopsy at the request of the owners.

### Autopsy and laboratory findings

**Fig. 1.**
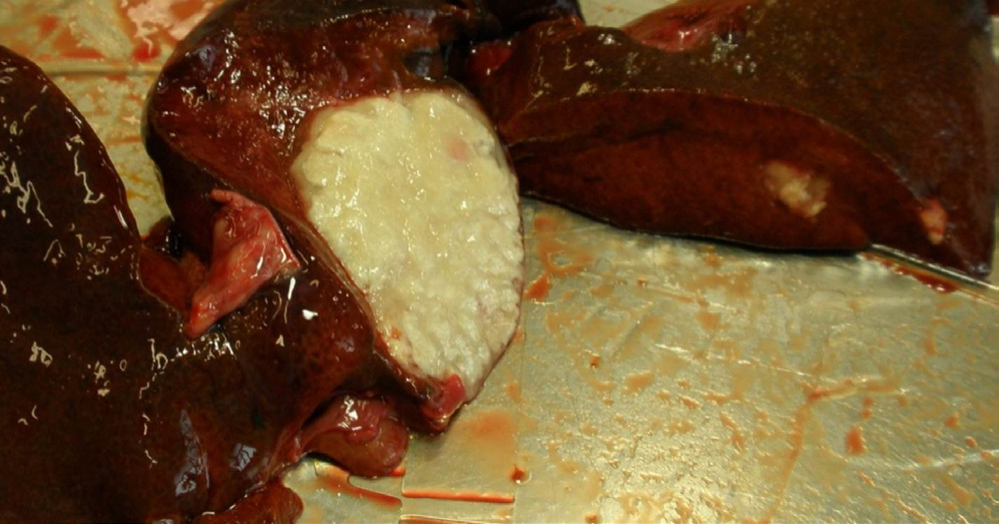
Cut surface of liver granulomas showing typical caseotic structure. There was a very large necrotic granuloma, and few smaller granulomas were scattered throughout the other liver lobes.

**Fig. 2.**
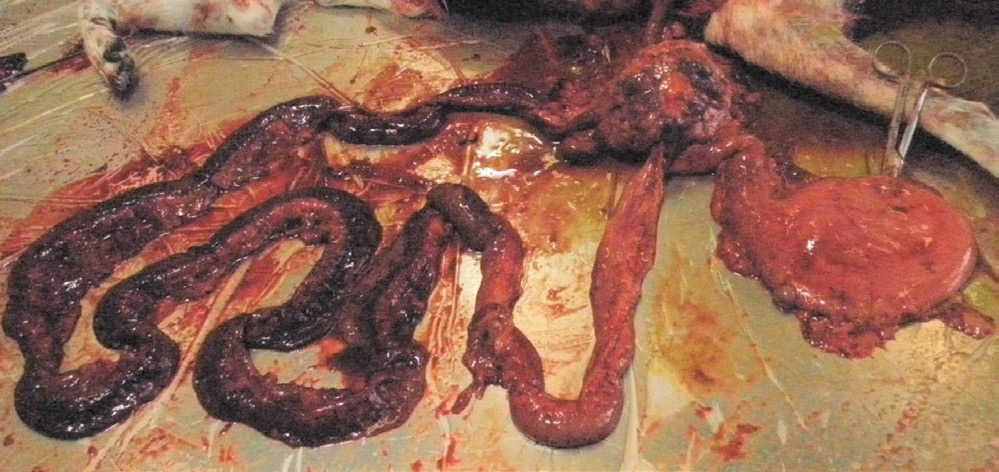
There were marked chronic inflammatory lesions in the intestines and the small bowel was perforated.

Autopsy was performed at the Finnish Food Authority, Production and Companion Animal Pathology Section. Opportunistic yeast infection in the liver was confirmed and severe large chronic inflammatory granulomatous lesions were detected in the intestinal wall, mesenteric lymph nodes, liver and kidneys. There was one very large necrotic granuloma in the right lateral liver lobe, and a few smaller granulomas were scattered throughout the other lobes (Fig. 1). The granulomas showed a typical caseotic structure. The small intestine was acutely perforated causing septic peritonitis (Fig. 2). In addition, granulomatous interstitial pneumonia was recorded. Culture specimens were collected from the most affected sites, the liver, the mesenteric lymph nodes and the intestine, and *M. tuberculosis* was isolated on Löwenstein-Jensen, Coletsos and Coletsos Ossein (Bio-Rad) media from all collected specimens at the Finnish Food Authority, Bacteriology Section. Initial identification was done using an AccuProbe Mycobacterium tuberculosis complex kit (Gen-Probe) following the manufacturer’s instructions.

Identification of *M. tuberculosis* was confirmed in the National Mycobacterial Reference Laboratory by using a Hain Lifescience Genotype MTBC kit according to the manufacturer’s instructions using a SensoQuest Labcycler for PCR for the liver and intestinal isolates. Both isolates were fully susceptible by molecular testing using a Hain Genotype MTBDR PLUS kit (rifampicin, isoniazid), via a culture-based method using the Becton Dickinson MGIT system (streptomycin 1.0 µg ml^−1^, isoniazid 0.1 µg ml^−1^, rifampicin 1.0 µg ml^−1^, ethambutol 5.0 µg ml^−1^, pyrazinamide 100 µg ml^−1^) and by whole-genome sequencing. The sequencing pipeline included a Qiagen MagAttract HMW DNA extraction kit, Illumina Nextera XT library preparation kit, Beckman Coulter Agencourt Ampure XP magnetic bead purification, Illumina MiSeq Reagent Kit v2 on the Illumina MiSeq sequencing platform (according to the manufacturer’s instructions), and PhyResSE web tool (http://www.phyresse.org/) as described elsewhere [15, 16]. The isolates belonged to lineage Ural (PhyResSE, SITVIT2), and their spoligotype was SIT262 by Spotyping analysis [17, 18].

## Discussion

The import of companion animals carrying communicable diseases represents a public health risk of which the general public is usually not aware. The import of rescue animals at the present extent is a relatively new phenomenon and is increasing heavily. Given the number of reports of single tuberculotic canine cases with striking similarities in the background data [6–11], it is reasonable to think that there are numerous unidentified cases among imported dogs in a repeating pattern that have gone unnoticed. In addition to the owners, vulnerable individuals and other pets that may be predisposed to lengthy exposure periods, the work safety of the personnel at veterinary clinics, and even more so the pathologists and personnel at autopsy are of highest importance, because they have an elevated risk of infection while working in close contact with diseased animals.

Compared with numerous other canine-associated severe and significant zoonootic diseases, such as rabies, echinococcosis, leishmaniosis, leptospirosis, listeriosis, ESBL, salmonellosis, giardiasis and vector-borne diseases [2, 19, 20], TB may be rare, but it is also more easily overlooked. Most of these infections have a faster progress and more prominent manifestations than TB, and may thus be recognized more easily in a timely manner. It may take years from TB infection to clinical disease. In dogs, the initial stages of the active phase are asymptomatic and TB may be have progressed before owners notice any signs [21]. In later stages, the clinical signs are non-specific and the origin of the dog is not recognized as the source of infection. In the present case, several factors indicate that the infection probably originated from Romania rather than Finland. The disease was progressive, disseminated and chronic, suggesting a non-recent time of infection, there was a locally rare yeast co-infection, the incidence of TB is 14.5 times higher in the originating country, and there was no history of TB in the present family. The genotype did not confirm the origin, as the Ural genotype is globally uncommon (<15 %) and mostly detected in Central Eurasia, i.e. Russia and former Soviet Union countries [22].

The dog in the present case was known to have lived in an urban area next to a hospital before capture, and thus the infection may have originated from eating clinical waste. There were no data regarding the previous owner. The dog was, however, asymptomatic for 2 years after import and considered healthy, so the origin of the gastrointestinal symptoms was not suspected to be from abroad. The disease progress was influenced by cortisone medication, prescribed due to suspicion of food hypersensitivity. Cortisone decreases inflammation and reduces the activity of the immune system. Acid-fast bacilli were recorded from biopsies taken on laparotomy but no further identification was performed at that stage. The dog was symptomatic, i.e. diarrhoeic and vomiting due to intestinal TB, and thus it was potentially infectious for 9 months while diagnosis was delayed until autopsy, and the disease had disseminated to several organs. Additionally, histoplasmosis co-infection may have contributed to the symptoms and intestinal signs, given that intestinal histoplasmosis may also cause persistent diarrhoea and ulceration of the intestinal tract. Because TB is a rare disease in Finland, the possibility of TB may be overlooked, or the disease misdiagnosed, in both human and animal healthcare, even with suggestive clinical signs.

The owners of the present case were exposed to vomit and diarrhoeal and faecal material during the symptomatic period of January–October 2018. Contact tracing of the owners was performed according to national guidelines, including chest X-ray and medical examination at 3 and 12 months after last exposure; an IGRA (IFN gamma release assay) was not performed due to the age of the owners.

The children and grandchildren were present only occasionally, and were not handling vomit or faecal material, and thus despite petting and licking, the cumulative exposure time remained low. Other exposed persons included personnel present at laparotomy or autopsy, i.e. invasive procedures or handling infectious material or cleaning without proper respiratory protection. All exposed persons who were identified were notified of the exposure and the symptoms of the disease, and none have had symptoms so far. Exposed animals in dog parks were not traced.

Similar to the present case, an uncommon enteric clinical case of TB in a beagle dog with intense intestinal infiltration was reported in Brazil [9]. In that case the primary route of infection was suspected to be through contaminated homemade food containing tubercle bacilli excreted by a TB‐positive owner. In France a boxer dog with vomiting, diarrhoea and weight loss had previously moved with the owners from West Africa, and died with disseminated pleural TB [7]. The source was believed to be the family’s African cook who was in close contact with the dog, and died without a diagnosis, but with TB-like symptoms.

Although treatment of TB in a pet is not often an option, a rare successful treatment of intra-abdominal TB in a mixed-breed dog originating from Greece was reported in Germany [23]. Treatment required extended medical follow-up of the dog and dedicated education of the owners. In Italy, five indoor cats in a cattery with respiratory *M. bovis* infection were euthanized despite treatment [3]. Import of a kitten that had a bite wound from Ukraine was the suspected source. Although the risk for dog-to-owner transmission is considered very low, the risk for dog-to-dog transmission is higher, as seen in the fulminant *M. bovis* outbreak among English foxhound dogs in a hunt kennel, where 52 % of 164 dogs were IGRA-positive and almost 10 % became clinically ill [24].

The chance that autopsy is performed for a family dog dying of an unknown cause is fairly high in high-income countries. The risk to autopsy personnel is plausible and real. Three veterinary pathologists in Switzerland became infected with *M. tuberculosis* through direct contact while performing an autopsy for a severely infected Ibizan hound dog, originally a stray dog from southern Europe, with infection focus in the central nervous system [25]. The pathologists tested IGRA-positive but had no clinical signs. The owners and clinical personnel remained negative, except for one clinician who had another more likely source of exposure. Additionally, one of the two cats living in the same household with the infected dog was IGRA-positive.

Dogs are the most popular companion animals, with around 85 million pet dogs in Europe (www.statista.com), and their numbers are increasing. More and more dogs are being imported and the number of companion animals carrying communicable diseases across borders is likely to rise. People and the environment are protected by official requirements for importing animals, such as EU Pet Travel Scheme. The minimum requirements are identification by a microchip or a tattoo, vaccination against rabies, echinococcus treatment and pet passport. The Pet Travel Scheme allows free movement of people and their pets, i.e. up to five dogs. So pet dogs accompanying holidaymakers may present a similar risk [19]. Illegal import, such as puppy smuggling, or unintentional import, such as adopting a stray dog during a caravan holiday, are largely beyond control. Better awareness of the risks associated with small-scale import and alertness at border control or more stringent control mechanisms are needed [19].

In contrast to pulmonary *M. bovis, M. tuberculosis* has not been shown to transfer from animals to humans in everyday contact, although the organism is present in dog respiratory excretions. In Finland, there is one known case of a pregnant woman with TB in which the suspected source was her dog that was in contact and possibly ate sputum excreted by homeless alcoholics staying in the park where the dog was walked regularly (Dr Hanna Soini, pers. comm.). As with imported human TB [26], the overall impact of imported canine *M. tuberculosis* on public health in low-incidence countries is low, but at an individual level, the consequences of an infection are dramatic and the costs to healthcare and infectious disease surveillance systems are surprisingly high. The risk of an infection is elevated in individuals with reduced immunity and in children, and the consequences are more dramatic in cases with multidrug-resistant *M. tuberculosis* [27].

With regard of possible human health risks, it is of upmost importance to be aware of the origins of a severely ill imported dog. Homeless dogs should be helped, but the most effective, safe and risk-free way to do it is to help animals in their own home countries. TB may be suspected when a dog has non-specific persistent clinical signs with potential exposure to TB-infected owners or cattle, or if the animal originates from a high-incidence TB country. Disease awareness through accessible information to both the general public and veterinarians, availability of rapid diagnostics and proper personal protection remain crucial.
